# Efficacy and Safety of Arachidonic Acid for Treatment of School-Age Children in *Schistosoma mansoni* High-Endemicity Regions

**DOI:** 10.4269/ajtmh.14-0675

**Published:** 2015-04-01

**Authors:** Rashida Barakat, Nadia E. Abou El-Ela, Soraya Sharaf, Ola El Sagheer, Sahar Selim, Hatem Tallima, Maaike J. Bruins, Kevin B. Hadley, Rashika El Ridi

**Affiliations:** Tropical Health Department, High Institute of Public Health, Alexandria University, Alexandria, Egypt; Parasitology Department, National Liver Institute, Menoufiya University, Shebin El-Kom, Menoufiya, Egypt; Zoology Department, Faculty of Science, Cairo University, Cairo, Egypt; Research and Development Department, DSM Biotechnology Center, Delft, The Netherlands; Human Evidence Department, DSM North America, Columbia, Maryland

## Abstract

Arachidonic acid (ARA), an omega-6 fatty acid, is a potent schistosomicide that displayed significant and safe therapeutic effects in *Schistosoma mansoni*-infected schoolchildren in *S. mansoni* low-prevalence regions. We here report on ARA efficacy and safety in treatment of schoolchildren in *S. mansoni* high-endemicity areas of Kafr El Sheikh, Egypt. The study was registered with ClinicalTrials.gov (NCT02144389). In total, 268 schoolchildren with light, moderate, or heavy *S. mansoni* infection were assigned to three study arms of 87, 91, and 90 children and received a single dose of 40 mg/kg praziquantel (PZQ), ARA (10 mg/kg per day for 15 days), or PZQ combined with ARA, respectively. The children were examined before and after treatment for stool parasite egg counts and blood biochemical, hematological, and immunological parameters. ARA, like PZQ, induced moderate cure rates (50% and 60%, respectively) in schoolchildren with light infection and modest cure rates (21% and 20%, respectively) in schoolchildren with high infection. PZQ and ARA combined elicited 83% and 78% cure rates in children with light and heavy infection, respectively. Biochemical and immunological profiles were either unchanged or ameliorated after ARA therapy. Combination of PZQ and ARA might be useful for treatment of children with schistosomiasis in high-endemicity regions.

## Introduction

Schistosomiasis is a severe parasitic disease of man caused by flatworms of the genus *Schistosoma*. *S. mansoni* and *S. haematobium* are responsible for the majority of human infections, which start with skin penetration by the cercariae shed by specific freshwater snails. In the epidermis, the invading larvae transform into schistosomula. Biochemical and physiological changes include replacement of the larva trilaminate outer membrane with a sphingomyelin (SM)-rich double-lipid bilayer and presumably, as a consequence, protection by a hydrogen bond barrier before entering blood capillaries for permanent intravascular residence.^1^,^2^ The eggs deposited by the adult female worms burrow through the wall of the blood vessels to the gut or bladder lumen and exit through feces or urine or are retained in the host tissues, inducing intense inflammatory reactions that are responsible for the majority of the disease symptoms.^3^ The sequelae are grave and reflected in more than 3.3 million disability-adjusted life years (DALYs) and remarkably high rates of years lived with disability (YLDs).^4^,^5^

Exposure to the infective stage is especially common among children in rural areas of developing countries. Other high-risk groups include farmers, fishermen, and people using infected water for their daily tasks. Because of the insensitivity of diagnostic techniques and the focal distribution of the infection, the World Health Organization (WHO) no longer provides estimates of the people infected or at risk worldwide. Instead, the WHO provides estimates of the population requiring preventive chemotherapy based on the risk of infection in the areas where they live that is based on the prevalence of infection in a sample of school-age children.^6^ Schistosomiasis prevalence level and infection intensity are, thus, determining eligibility factors for preventive chemotherapy with praziquantel (PZQ), which is currently the drug of choice for treatment of schistosomiasis. Opposite the data reported for some high-endemicity areas in Brazil, Mauritania, and Tanzania,^7^ schistosomiasis prevalence level seems to be an important factor regarding the outcome of PZQ chemotherapy. A recent meta-analysis of comparative and non-comparative clinical trials indicated that overall PZQ efficacy for *S. mansoni* is 76.7%.^8^ However, in Wondo Genet, southern Ethiopia, a region with a school children prevalence rate of 75% (i.e., a high-risk community), a single oral dose at 40 mg/kg body weight induced only 60% cure and egg reduction rates in 10- to 14-year-old children.^9^ In a study performed along the high-endemicity shores of Lake Victoria involving 178 *S. mansoni*-infected men, the cure rate after a single PZQ dose ranged from 36% to 82%.^10^ Exceptionally low PZQ cures were reported in children living in the Senegal Basin River after the construction of a dam that caused intense *S*. *mansoni* and *S. haematobium* transmission.^11^,^12^ The efficacy of PZQ, thus, seemed to depend on the rate and intensity of transmission.^9^–^16^ Repeated treatments were, therefore, recommended, increasing the threat of development of parasite resistance to the drug.^15^–^17^ The development of alternative treatments seems, thus, to be a priority. In that regard, it is crucial to assess the therapeutic effects of novel potential schistosomicides in low- and high-infection risk communities.

Arachidonic acid (ARA), which is a normal constituent of cell plasma membranes and the human diet, was found to *in vitro* and *in vivo* kill *S. mansoni* and *S. haematobium* juvenile and adult worms.^18^,^19^ The proposed ARA killing mechanism was activation of parasite surface membrane-associated neutral sphingomyelinase (nSMase) with consequent apical bilayer SM hydrolysis and disruption of the SM-based hydrogen barrier shielding the worm from the hostile elements of the immune system.^1^,^2^,^18^–^22^ We have recently reported on the efficacy and safety of ARA in treatment of school-age children residing in regions of low-endemicity *S. mansoni*.^22^ This study reports on the efficacy and safety of ARA alone or combined with PZQ in treatment of school-age children residing in *S. mansoni* high-endemicity areas.

## Materials and Methods

### Ethical considerations.

The study protocol was reviewed and approved by the Ethical Committee of the Egyptian Ministry of Health and Population, Central Directorate for Research and Health Development (Approval 34-2012/1). The study was registered with ClinicalTrials.gov (NCT02144389). Written informed consent was obtained from parents or legal guardians of all children enrolled in this study after explaining the objectives and methods of the study.

### Drugs.

PZQ tablets (Distocide; Epico Pharmaceuticals, El-Asher-Men-Ramadan City, Sharqia, Egypt) were provided by the Egyptian Ministry of Health and Population. ARA oral capsules containing 396 mg ARA (ARASCO) per capsule were provided by DSM Nutritional Products (Columbia, MD). Placebo (Pbo) capsules lacking ARA and containing a corn–soybean oil blend were used as control for children treated with PZQ only.

### Study design.

#### Subjects selection.

**Figure 1.**
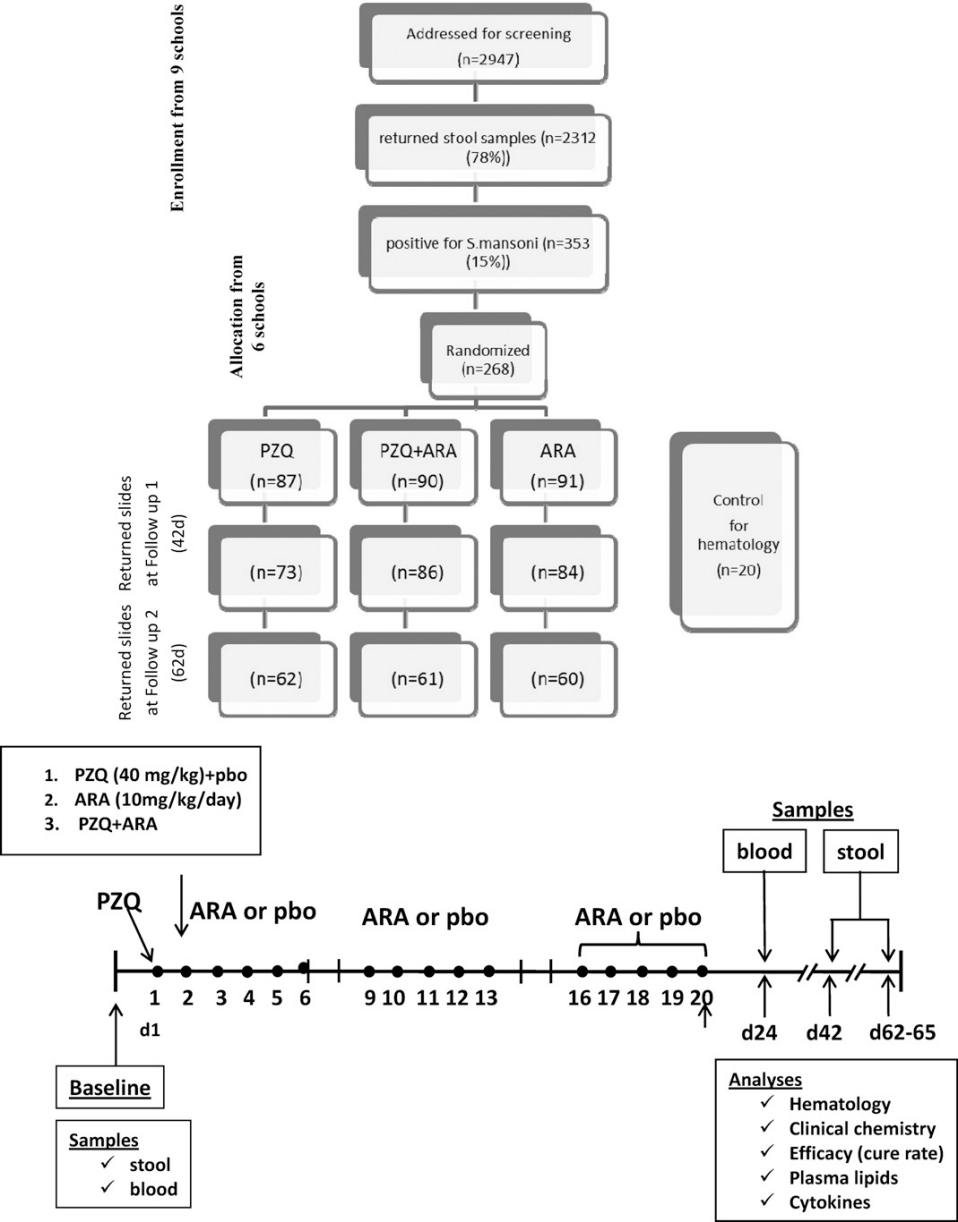
Study flow chart. Only 268 of 353 positive children were included in the study based on the availability of ARA capsules shipped from DSM in Columbia, MD to Egypt.

The study was carried out between October of 2012 and May of 2013, and it was conducted in two phases. Phase I was screening. All of the 2,947 students (1,549 boys [52%] and 1,398 girls [48%]) in grades III–V from nine primary schools in Motoubis area, Kafr El Sheikh Governorate were included in the screening phase. Only 2,312 children (78%) provided stool samples (1,181 boys [51%] and 1,131 girls [49%]). For each child, two microscopic slides of stool samples on 3 consecutive days were examined for egg counts per gram of stool (epg) by Kato–Katz method as described.^23^,^24^ In total, 353 children (15%) were positive (215 boys [61%] and 138 girls [39%]). Only 268 of 353 positive children were included in the study based on the availability of ARA capsules shipped from DSM to Egypt, and they were stratified according to the intensity of infection into light (< 100 epg), moderate (100–400 epg), and heavy (> 400 epg) infection. Of note, 13, 14, and 7 schoolchildren with light, moderate, and heavy *S. mansoni* infection, respectively (total of 34 of 268 = 13%), additionally suffered light infection with *Ascaris lumbricoides* and/or *Ancylostoma duodenale*. Phase II was the randomized clinical trial's Pbo-controlled phase. In total, 268 schoolchildren attending six schools in three villages were randomly assigned to three treatment groups (PZQ + Pbo, ARA, or PZQ + ARA) using computerized random selection. Per stratum, the children were proportionally allocated based on intensity of *S. mansoni* infection to three treatment groups, where approximately 45% had light infection level and the rest had moderate or heavy infection levels based on egg counts. In total, 160 males and 108 females (9-to 11-year-old children with weight ranges of 20–50 kg) participated in the randomized Pbo-controlled study (Figure 1).

#### Treatment.

The first group of selected children was given a single oral dose of PZQ (40 mg/kg) on the first day of treatment and Pbo oil capsules for the next 3 weeks (five doses per week). The second group orally received ARA (10 mg/kg per day) for 15 days over 3 weeks (5 days/week). The third group was given PZQ (40 mg/kg) on the first day of treatment and then, received 15 doses of ARA (10 mg/kg per day for 5 doses/week). The total numbers of children were 87, 91, and 90 for PZQ, ARA, and combined PZQ and ARA study arms, respectively. All children were monitored daily by experienced medical personnel, and data were gathered through a questionnaire enquiring on the occurrence of adverse reactions.

#### Analyses.

Approximately 10 mL blood was obtained by venous puncture from each child 2–3 days before the start of treatment and 3 days after the end of ARA (or ARA + PZQ) treatment, corresponding to 24 days after PZQ treatment. In total, 20 sex-, age-, and social conditions-matched, parasite-free children were considered as controls. The blood was aseptically and equally distributed among four vacutainers that were plain or contained sodium citrate, ethylene diamine tetraacetic acid disodium salt (EDTA), or sodium heparin (BD; Becton, Dickinson and Company, Plymouth, United Kingdom) and transported within 3 hours at 10°C to the laboratory. Sera and plasma were immediately frozen at −76°C. For the efficacy study, coded stool samples were obtained from children on 3 consecutive days 3 weeks after the end of ARA treatment (corresponding to 6 weeks after PZQ treatment) and 6 weeks after the end of ARA treatment, and epg was assessed by two technicians entirely blind to the code (Figure 1).

### Biochemical and hematological parameters.

Biochemical and hematological parameters were assayed for each child before and after treatment essentially as described previously.^22^ Serum was used to investigate the lipid profile and parameters reflecting liver function, alanine aminotransferase (ALT), aspartate aminotransferase (AST), and bilirubin as well as creatinine reflecting kidney function (all by Modular P1600; Roche Diagnostic, Hitachi P; Germany). Viral markers, including both hepatitis B surface antigen and hepatitis C virus antibody, were analyzed using Architect i2000SR (Abbott Diagnostics, Abbott Park, IL) and found to be uniformly negative. Blood samples in tubes containing sodium citrate were used for analysis of coagulation tests, including prothrombin time and activated partial thromboplastin time, by an automated blood coagulation analyzer (STA Compact; Diagnostica Stago, Paris, France); 2 mL blood dispensed in EDTA tubes was analyzed for complete blood chemistry by using an automated blood hematology analyzer (Beckman Coulter LH785; Beckman Coulter, Miami, FL).

Aliquots of heparinized plasma collected at baseline and after treatment were stored at −76°C and analyzed for levels of total lipids and unsaturated fatty acids (FAs) as described.^22^

### Levels of plasma interleukin-10 and interferon-γ.

Plasma was retrieved from heparinized blood after centrifugation at 400 × *g* for 20 minutes and stored at −76°C until assayed by capture enzyme-linked immunosorbent assay (ELISA) for levels of interleukin-10 (IL-10) and interferon-γ (IFN-γ; ELISA MAX Set; BioLegend, San Diego, CA) according to the manufacturer's protocol.

### Whole-blood cultures cytokine response to parasite antigen.

Heparinized whole-blood samples were diluted 1:4 in culture medium as described,^22^ and 200 μL were incubated in duplicate with 50 μL medium containing 0 or 100 μg/mL soluble adult worm antigen (SAWA) prepared as described previously.^25^ Whole-blood cell cultures were incubated for 72 hours at 37°C and 3% CO_2_ and then centrifuged at 400 × *g* for 10 minutes. The cell-free supernatants were transferred into wells of sterile plates and stored at −76°C until assayed by capture ELISA for levels of released IL-4 (or IL-5), IL-17, and IFN-γ (ELISA MAX Set; BioLegend) following the manufacturer's instructions.

### Statistical analysis.

All values were tested for normality. Student's independent two-tailed *t*, Mann–Whitney, χ^2^, and Fisher exact tests were used to analyze data. Statistical significance was set at *P* < 0.05.

## Results

### Efficacy of ARA treatment.

The efficacy of ARA in treatment of schoolchildren with light infection (< 100 epg) was highly comparable with that of PZQ + Pbo, with percentage cure of 50% and 60%, respectively, but highly different levels of reduction in geometric mean egg counts (GMEC) in uncured children. Therapeutic efficacy of PZQ + ARA reached 83%, which was significantly (*P* = 0.0005, Fisher exact test) higher than 60% for PZQ alone (Table 1). The efficacy of ARA in treatment of schoolchildren with moderate infection (100–400 epg) was significantly (*P* < 0.0001) lower than for PZQ regarding cure rates of 23 and 26 children, respectively, and reduction in GMEC in uncured children. For these moderately infected children, ARA again showed significant (*P* < 0.05) additive effect to PZQ, resulting into 57% compared with 42% cure (Table 1). Efficacy of ARA and PZQ was quite comparable in school-aged children with heavy infection (800–1,000 epg), leading to 21% and 20% cure rates, respectively, and 64% and 81% reductions in GMEC in uncured children, respectively. The combination of PZQ and ARA resulted in a highly significant (*P* < 0.0001) increase in cure rates (78%) compared with 20% for PZQ alone but not egg reduction rates (Table 1).

### Safety of ARA treatment.

Not a single child reported the slightest adverse reaction during or after treatment with ARA. Adverse reactions after PZQ treatment were similar to those reported for Menoufiya children.^22^

#### Lipid profile.

Therapy of *S. mansoni* patently infected schoolchildren with PZQ, ARA, or PZQ and ARA combined did not affect the serum levels of cholesterol and high- and low-density lipoproteins (Supplemental Table 1) or the plasma concentration of total saturated FAs, monounsaturated FAs, or n-3 polyunsaturated FAs (Supplemental Table 2). However, mean weight percentage of ARA in plasma was highest after treatment with ARA only or combined with PZQ compared with levels at baseline and post-treatment with PZQ. Twenty-four days after treatment with PZQ, a statistically relevant decrease in total saturated FA was observed compared with baseline. Treatment-related differences in ARA levels in plasma were mirrored by minor but statistically significant reductions in the precursor for ARA, linoleic acid (18:2n-6). Docosahexaenoic acid levels in plasma were not affected by treatment (Supplemental Table 2).

#### Liver functions.

All school-age children's values for ALT, AST, and bilirubin were in the normal range before and after therapy (Supplemental Table 3).

#### Kidney functions.

Therapy with ARA led to a significant (*P* < 0.05) decrease in levels of plasma creatinine (Supplemental Table 4).

#### Hematology.

Treatment with PZQ, ARA, or PZQ and ARA combined of *S*. *mansoni*-infected schoolchildren failed to significantly affect the number of erythrocytes, packed cell volume percentage, hemoglobin concentration (data not shown), and levels of eosinophils, basophils, segmented neutrophils, and platelets (Table 2).

#### Coagulation parameters.

The three treatment regimens were similar regarding their effects on *S. mansoni*-infected schoolchildren's blood-clotting parameters (Table 3).

### Effects of ARA treatment on immunological parameters.

**Figure 2.**
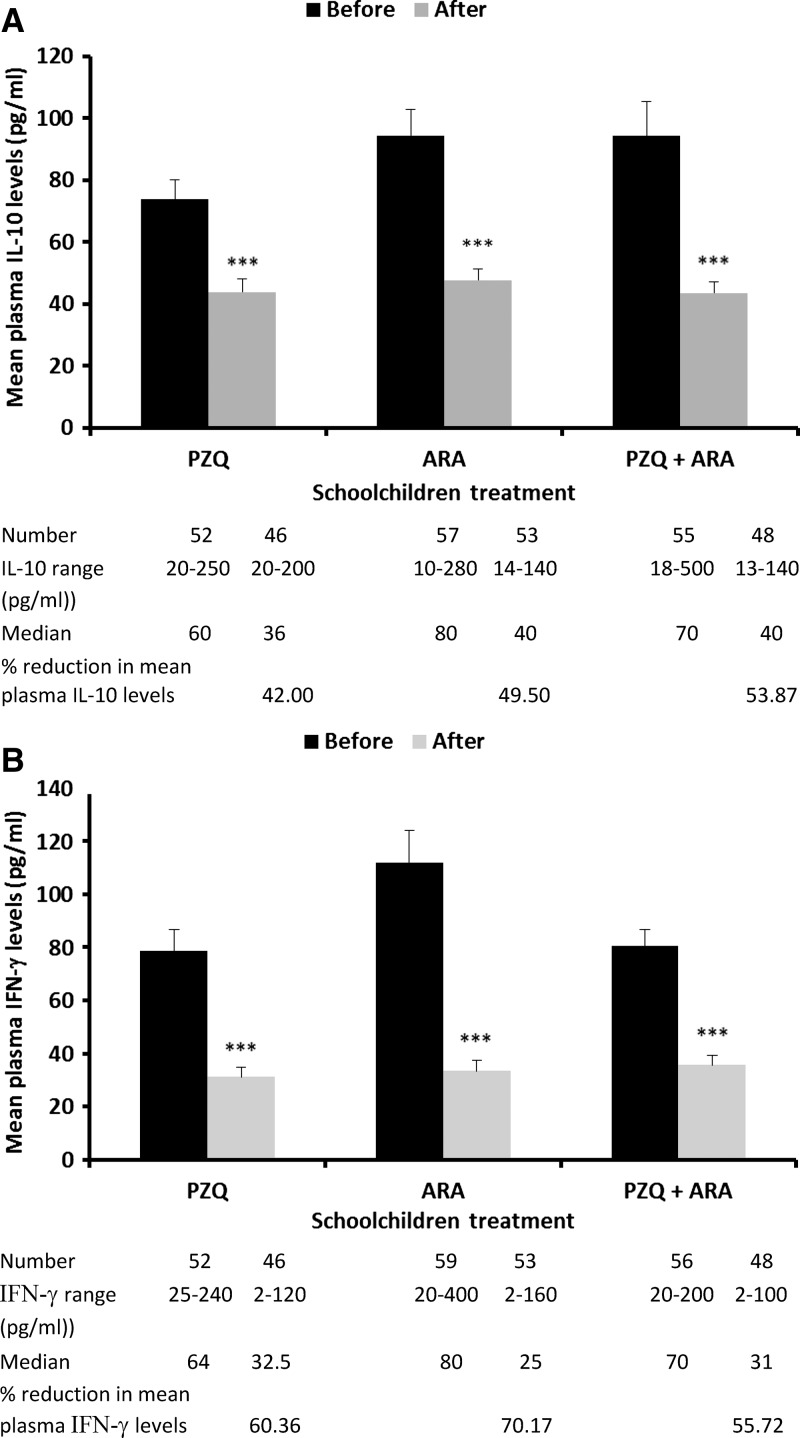
Effect of ARA treatment on levels of IL-10 and IFN-γ in plasma of schoolchildren infected with *S. mansoni* and treated with PZQ, ARA, or PZQ + ARA. Columns represent mean plasma (**A**) IL-10 and (**B**) IFN-γ levels, and horizontal bars denote the SEM. Percentage reduction = mean cytokine level before treatment – mean cytokine level after treatment/mean cytokine level before treatment × 100 evaluated for each child 3 days after the end of ARA treatment corresponding to 24 days after PZQ. ***P < 0.0001 as calculated using paired and unpaired two-tailed Student's *t* test versus baseline.

All 20 sex- and age-matched parasite-free children had no detectable circulating IL-10 levels (data not shown), whereas 164 patently infected children had 20–500 pg/mL IL-10. Children with light, moderate, and heavy infections had baseline plasma IL-10 levels of 93 ± 75, 88 ± 61, and 80 ± 51 pg/mL, respectively (mean ± SE for 73, 50, and 41 children, respectively), with no significant differences related to intensity of *S. mansoni* infection or affliction with *Ascaris* or *Ankylostoma*. Treatment with PZQ, ARA, or PZQ combined with ARA led to a highly significant (*P* < 0.0001) decrease of 42–54% in IL-10 levels (Figure 2A), again regardless of baseline infection intensity. In total, 167 schoolchildren patently infected with *S. mansoni* had 20–400 pg/mL plasma IFN-γ, far above the level of approximately 30 pg/mL detected in sex- and age-matched parasite-free children (data not shown). Children with light, moderate, and heavy infections had baseline IFN-γ levels of 107 ± 76, 74 ± 55, and 88 ± 76 pg/mL, respectively (mean ± SE for 71, 50, and 46 children, respectively), with no significant differences related to intensity of *S. mansoni* infection or occurrence of other parasites. Treatment with ARA alone led to the highest decrease in levels of circulating IFN-γ (Figure 2B) and the highest increase in percentage (84%) of children displaying decrease in IFN-γ levels compared with children treated with PZQ (78%) or PZQ + ARA (81%).

**Figure 3.**
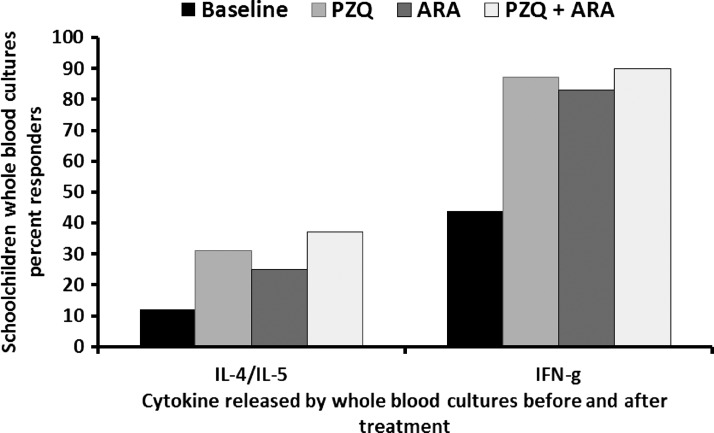
Effect of ARA treatment on whole-blood culture cytokine responses of schoolchildren infected with *S. mansoni*. Columns represent the percentage of schoolchildren with whole-blood cultures responding to *in vitro* SAWA stimulation by release of type 2 (IL-4/IL-5) or type 1 (IFN-γ) cytokine before (baseline; 262 children) or after treatment with PZQ (84 children), ARA (90 children), or PZQ + ARA (87 children).

Negligible levels of cytokines were detected in unstimulated or antigen-stimulated *ex vivo* whole-blood cultures of parasite-free donors and unstimulated cultures of patently infected children. At baseline before treatment, 12%, 20%, and 44% of SAWA-stimulated whole-blood cultures from a total of 262 *S. mansoni*-infected children released IL-4/IL-5, IL-17, and IFN-γ, respectively. At 3 days after the end of ARA therapy (corresponding to 24 days after the end of PZQ administration), 26 of 84 (31%), 23 of 90 (25%), and 32 of 87 (37%) children administered with PZQ, ARA, and PZQ + ARA, respectively, produced IL-4/IL-5 in response to SAWA. The increase in the percentage of responders with type 2 cytokine was significantly (*P* = 0.0017, *P* = 0.027, and *P* = 0.0001 for the PZQ, ARA, and PZQ + ARA study arms, respectively) higher compared with baseline value. No changes in percentage of IL-17 responders were observed after therapy compared with baseline. Conversely, the increase in percentage of IFN-γ responders was highly significant (*P* < 0.0001) for the three study arms compared with values before treatment, with 87%, 83%, and 90% of schoolchildren given PZQ, ARA, or PZQ + ARA, respectively, now responding by IFN-γ production to SAWA stimulation of whole-blood cells (Figure 3).

## Discussion

The key result of this study is that the efficacy of PZQ and ARA in treatment of schoolchildren's schistosomiasis is affected by the endemicity levels and intensity of mass PZQ treatment campaigns in the areas of residence. Thus, the efficacy of PZQ in treatment of school-age children residing in the schistosomiasis high-endemicity Motoubis area in Kafr El Sheikh^15^,^26^–^28^ and the low-prevalence Menouf district, Menoufiya^22^ vastly differed, with cure rates of 60% and 85% for children with low-intensity infection and 42% and 83% for children with moderate infection, respectively. Cure and egg reduction rates of approximately 58% were also recorded 6 weeks after PZQ treatment of school-age children with light and moderate infections residing in *S*. *mansoni* high-prevalence areas in Niger.^29^ In this study, PZQ cure rates of Kafr El Sheikh children with high-intensity infection assessed 6 weeks after treatment did not exceed 20%, a finding of considerable concern. The results are in accord with numerous research articles reporting association between PZQ efficacy and baseline infection intensity as well as levels of schistosomiasis prevalence and endemicity.^12^–^16^,^29^

Of note, in a study performed in 2009 and 2010, PZQ-mediated cure rates in school-age children in the *S. mansoni* high-endemicity El Rouse village in Kafr El Sheikh were 83%, 76%, and 54% for light, moderate, and heavy infection, respectively, 4 weeks after a single PZQ dose of 40 mg/kg.^15^ These cure rates are distinctly higher than those obtained in this study in the same governorate at 6 weeks after PZQ therapy, suggesting reduction in PZQ efficacy 3 years later. The alarming reduction in PZQ efficacy may be attributed to development of parasite resistance to the drug after intensive and repeated mass administration campaigns.^17^,^30^,^31^ However, the reduction as well in ARA-mediated cure rates (50% versus 78% and 13% versus 44% for lightly and moderately infected schoolchildren of Kafr El Sheikh and Menoufiya, respectively) may certainly not be attributed to resistance consequent to extensive use. The decrease in the efficacy of both PZQ and ARA may be attributed to differences in *S. mansoni* strains in the two governorates. Because massive PZQ mass treatment was applied for several consecutive years in Kafr El Sheikh Governorate, we propose that the decrease in PZQ and ARA efficacy may also be explained if repeated and intensive PZQ use selects for the worms with tighter outer lipid bilayer barriers consequent to higher percentages of cholesterol and SM and/or less-active tegument-associated nSMase. The result would be worm progeny able to prevent or decrease access of molecules of 312 Da, such as PZQ. Schistosomes showing PZQ insusceptibility were documented to incur serious biological costs,^31^ a strong support to our assumption. ARA at the plasma concentration achieved would fail to activate the parasite tegument-associated nSMase to the level necessary for worm attrition but would force openings in the outer lipid bilayer that allow PZQ to penetrate and exert its schistosomicidal action.^1^,^2^,^18^–^22^,^32^,^33^ PZQ sublethal effects may also elicit changes in the worm upper lipid bilayer, facilitating ARA action. The high cure rate achieved after use of PZQ and ARA combined provides circumstantial evidence for our proposition. We are currently examining this hypothesis through evaluation of the cholesterol/SM content and nSMase activity in worms derived from cercariae obtained from communities of low prevalence/low PZQ use and high endemicity/intense PZQ administration. If our hypothesis proves correct, then the mode of action of ARA makes it a candidate schistosomicide for promoting PZQ efficacy in treatment of children with high infection in *S*. *mansoni* highly endemic regions.^34^–^36^ Wise and thorough planning should lead to devising a schedule and regimen of ARA + PZQ treatment that would achieve the sorely required goal of 100% cure of all patients, regardless of their intensity baseline or community endemicity level.

ARA is the precursor of two compounds related to platelet function, thromboxane and prostacyclin,^37^ and was reported to cause platelet aggregation in adults given 6 g ARA/day for 3 weeks.^38^ However, approximately 0.5 g ARA daily intake for 15 days over 3 weeks did not alter school-age *S. mansoni*-infected children's platelets counts or blood coagulation parameters, which is in accordance with findings in schoolchildren of Menoufiya^22^ and healthy adults given 1.7 g ARA daily for 50 consecutive days.^39^ The safety of ARA in treatment of school-age children of diverse infection intensity in the high-endemicity area of Motoubis, Kafr El Sheikh was clearly reflected in preservation of liver and kidney functions. Additionally, ARA treatment did not impair the serum or plasma levels of saturated or monounsaturated FAs, cholesterol, and high- and low-density lipoproteins, confirming data reported for schoolchildren in the Menoufiya area^22^ and athletes given 1.0 g ARA daily for 50 days.^40^ Plasma level of ARA was increased in parallel with linoleic decrease, which was observed in adults receiving up to 3.6 g/day ARA for 14 days,^41^ with no harmful effects on blood cell differential counts or any other metabolic panel.

School-age children residing in schistosomiasis high-prevalence regions in Egypt displayed higher baseline levels of plasma IL-10 than children in low-prevalence regions (mean ≥ 80 versus 20 pg/mL, respectively), perhaps because of more intensive exposure to worm excretory–secretory products,^42^ whereas the differences in circulating IFN-γ baseline levels were less striking. Treatment with PZQ and/or ARA led to normalization of plasma IFN-γ levels and a highly significant (*P* < 0.0001) decrease in IL-10 levels, thus rendering the immune profile of the children nearer to normal, despite incomplete cure and persistence of ova in stool. The decrease in the plasma levels of the immunosuppressive IL-10 after PZQ and/or ARA treatment correlated with an increase in the percentage of blood cell cultures responding to stimulation with parasite antigens by production of IFN-γ. More importantly, a substantial proportion of children's *ex vivo* blood cell cultures released type 2 cytokines in response to parasite antigen stimulation, additional evidence for the increase in human type-2 responsiveness to schistosome antigens after treatment.^43^–^46^ These findings are in accordance with the data documenting the ability of PZQ and ARA to enhance human immune responses.^43^–^50^

In conclusion, the reliance on a single drug (PZQ) is a risk, because resistance in schistosomes may develop, which is substantiated by lower responsiveness to the drug in highly endemic areas. Combining PZQ with the omega-6 FA ARA can achieve higher cure rates in children exposed to high infection loads compared with each of the compounds separately.

## Supplementary Material

Supplemental Tables.

## Figures and Tables

**Table 1:** Effect of ARA treatment on schoolchildren's parasitological parameters

Treatment/infection level	Number	Weight (kg)	Baseline GMEC ± SE	Cure rate	GMEC in uncured children ± SE (ERR)
PZQ					
Light	32	31.7 ± 5.4	38.7 ± 27.5	19/32 = 60%*	19.3 ± 13.9 (50.1%)*
Moderate	26	31.6 ± 5.0	226.1 ± 79.6	11/26 = 42%*	67.4 ± 73.2 (70.2%)*
Heavy	15	31.3 ± 4.6	979.5 ± 423.9	3/15 = 20%*	182.1 ± 203.2 (81.4%)*
ARA					
Light	24	30.3 ± 5.1	34.2 ± 27.1	12/24 = 50%*	35.1 ± 3.1 (−2.6%)†
Moderate	23	32.3 ± 4.8	195.6 ± 71.2	3/23 = 13%†	100.0 ± 54.0 (48.8%)†
Heavy	14	29.3 ± 5.1	961.3 ± 330.6	3/14 = 21%*	341.0 ± 168.6 (64.5%)‡
ARA + PZQ					
Light	23	31.7 ± 6.0	41.3 ± 27.8	19/23 = 83%†	20.0 ± 6.3 (51.5%)*
Moderate	23	31.7 ± 5.0	215.8 ± 81.0	13/23 = 57%‡	57.1 ± 43.2 (73.2%)*
Heavy	14	30.8 ± 5.9	805.6 ± 359.8	11/14 = 78%†	126.8 ± 65.1 (84.2%)*

Cure for each child was estimated based on the result of examination of two Kato slides (43.7 mg stool per slide) from each of three consecutive stool samples. Cure rate was calculated using the formula: number of cured (0 eggs) schoolchildren/number of treated schoolchildren × 100. Cure rates and egg reduction rates (ERRs; mean baseline GMEC – mean GMEC 6 weeks after treatment/mean baseline GMEC × 100) were evaluated 6 weeks after PZQ treatment of the PZQ group and 6 weeks after the end of ARA treatment of the two other groups. Groups of the same infection intensity and with different superscripts are significantly different as assessed by the Fisher Exact test.

**Table 2:** Effects of ARA treatment on blood granulocyte percentages and platelets counts of schoolchildren infected with *S. mansoni*

	Mean ± SD before and after treatment with		
	PZQ	ARA	PZQ + ARA
Children	84	89	85
Parameter (relative %)			
Eosinophils (%)			
Before	10.6 ± 6.4	10.9 ± 8.3	11.6 ± 9.3
After	10.7 ± 8.4 (NS)	11.2 ± 10.5 (NS)	11.9 ± 10.5 (NS)
Basophils (%)			
Before	0.5 ± 0.3	0.5 ± 0.4	0.5 ± 0.4
After	0.4 ± 0.3 (NS)	0.4 ± 0.3 (NS)	0.3 ± 0.3 (NS)
Segmented (%)			
Before	40.2 ± 10.6	39.9 ± 12.1	38.2 ± 13.3
After	39.4 ± 11.6 (NS)	38.5 ± 11.9 (NS)	38.2 ± 12.4 (NS)
Platelets counts			
Before	252,063 ± 64,374	254,213 ± 59,739	251,200 ± 56,305
After	253,154 ± 61,074 (NS)	246,244 ± 65,596 (NS)	244,081 ± 64,501 (NS)

Values before and after treatment were analyzed by the two-tailed paired Student's *t* test. NS = not significant.

**Table 3:** Effects of ARA treatment on blood-clotting parameters of schoolchildren infected with *S. mansoni*

	Mean ± SD before and after treatment with		
	PZQ	ARA	PZQ + ARA
Children	84	88	83
Parameter			
PT (seconds)			
Before	14.43 ± 0.96	14.25 ± 0.75	14.31 ± 0.71
After	13.98 ± 0.61	13.99 ± 0.52	14.01 ± 0.60
*P* value	0.0005	0.0122	0.0030
CT (seconds)			
Before	13.19 ± 0.02	13.19 ± 0.02	13.20 ± 0.36
After	13.09 ± 0.01	13.10 ± 1.04	13.10 ± 0.64
*P* value	< 0.0001	< 0.0001	NS
PT concentration (%)			
Before	84.62 ± 9.89	86.18 ± 8.45	85.49 ± 8.31
After	87.50 ± 7.82	87.32 ± 8.04	87.03 ± 7.71
*P* value	0.0375	NS	NS
INR			
Before	1.11 ± 0.09	1.10 ± 0.07	1.11 ± 0.07
After	1.08 ± 0.06 (NS)	1.08 ± 0.06 (NS)	1.09 ± 0.06 (0.035)
PT/INR			
Before	1.09 ± 0.07	1.07 ± 0.05	1.08 ± 0.05
After	1.06 ± 0.04	1.10 ± 0.31	1.10 ± 0.32
*P* value	0.0163	NS	NS
PTT (seconds)			
Before	34.38 ± 8.10	33.10 ± 4.21	33.15 ± 4.87
After	33.81 ± 4.57 (NS)	33.50 ± 5.19 (NS)	33.48 ± 5.19 (NS)

Prothrombin time (PT) and concentration, international normalized ratio (INR), and partial thromboplastin time (PTT) values before and after treatment were analyzed by the two-tailed paired Student's *t* test. CT = clotting time; NS = not significant.
