# Supplementary material for: Efficacy and Safety of Arachidonic Acid for Treatment of School-Age Children in Schistosoma mansoni High-Endemicity Regions
Source: Am J Trop Med Hyg. 2015 Apr 1;92(4):797–804. doi: 10.4269/ajtmh.14-0675 (PMC4385776; doi:10.4269/ajtmh.14-0675)
Supplement: Supplementary file 1 [file SD5.pdf]

Supplemental Table 1

Effects of ARA treatment on plasma lipid profile of schoolchildren infected with *S. mansoni*

	Mean ± SD before and after treatment with		
	PZQ	ARA	PZQ + ARA
Children	82	86	86
Parameter (mg/dL)			
Cholesterol			
Before	137 ± 27	140 ± 28	137 ± 28
After	139 ± 26 (NS)	139 ± 29 (NS)	137 ± 28 (NS)
Triglycerides			
Before	60.5 ± 26.1	65.7 ± 33.4	63.0 ± 31.8
After	71.5 ± 26.2	72.7 ± 28.7	68.1 ± 29.3
P value	0.019	NS	NS
High-density lipoprotein			
Before	42.3 ± 11.6	43.8 ± 11.9	45.5 ± 15.4
After	40.4 ± 9.2 (NS)	43.6 ± 10.7 (NS)	43.1 ± 10.9 (NS)
Low-density lipoprotein			
Before	81.2 ± 23.0	81.7 ± 23.2	77.1 ± 27.5
After	84.7 ± 23.8 (NS)	81.3 ± 26.0 (NS)	81.2 ± 24.6 (NS)

NS = not significant.

Supplemental Table 2

The FA composition of plasma in a subset of children from Kafr El Sheikh

	Total FAs (%) ± SD			
	Baseline	PZQ	ARA	PZQ + ARA
N	25	10	7	9
Total SFA	27.8 ± 1.5	26.8 ± 1.1$^{(0.03)}$	26.9 ± 1.1	27.1 ± 1.7
Total MUFA	18.9 ± 2.3	18.1 ± 2.5	18.0 ± 0.8	17.7 ± 1.8
PUFA				
<u>18:2n-6</u>	37.7 ± 3.0	38.3 ± 3.2	34.5 ± 3.8	35.4 ± 54.2
18:3n-6	0.6 ± 0.3	0.6 ± 0.2	0.6 ± 0.3	0.5 ± 0.1
20:2n-6	0.3 ± 0.1	0.3 ± 0.1	0.3 ± 0.0	0.3 ± 0.1
20:3n-6	1.8 ± 0.3	1.8 ± 0.2	1.4 ± 0.4$^{(0.01)}$	1.7 ± 0.3
<u>20:4n-6</u>	7.9 ± 1.3	9.4 ± 2.5	12.1 ± 1.8$^{(0.000)}$	12.5 ± 1.9$^{(0.000)}$
22:4n-6	0.3 ± 0.1	0.3 ± 0.2	0.4 ± 0.1$^{(0.03)}$	0.4 ± 0.1$^{(0.03)}$
22:5n-6	0.2 ± 0.1	0.2 ± 0.1	0.2 ± 0.2	0.2 ± 0.2
Total n-6 FAs	48.9 ± 3.0	50.4 ± 3.4	50.2 ± 1.7	50.9 ± 3.0
18:3n-3	0.6 ± 0.2	0.7 ± 0.2	0.6 ± 0.1	0.6 ± 0.1
20:5n-3	0.5 ± 0.5	0.6 ± 0.6	0.6 ± 0.6	0.6 ± 0.6
22:5n-3	0.5 ± 0.1	0.6 ± 0.1	0.6 ± 0.2	0.7 ± 0.2$^{(0.04)}$
<u>22:6n-3</u>	2.6 ± 0.4	2.7 ± 0.5	3.0 ± 0.5$^{(0.04)}$	2.8 ± 0.5
Total n-3 FAs	4.3 ± 0.5	4.6 ± 1.1	4.9 ± 0.8	4.3± 0.7

FAs levels were investigated in plasma of schoolchildren before (baseline) and after treatment with PZQ, ARA, or PZQ + ARA. Superscripts indicate *P* values for treatments versus baseline using two-tailed unpaired Student's *t* test. MUFA = monounsaturated FA; PUFA = polyunsaturated FA; SFA = saturated FA; 18:2n-6 = linoleic acid; 20:4n-6 = ARA; 22:6n-3 = docosahexaenoic acid.

Supplemental Table 3

Effects of ARA treatment on liver function parameters of schoolchildren infected with *S. mansoni*

	Mean ± SD before and after treatment with		
	PZQ	ARA	PZQ + ARA
Children	84	89	86
Parameter			
ALT (U/L)			
Before	13.7 ± 4.3	14.2 ± 6.3	14.1 ± 7.1
After	16.8 ± 6.6	16.0 ± 6.5	16.7 ± 9.6
P value	0.0006	NS	NS
AST (U/L)			
Before	22.2 ± 4.6	22.1 ± 8.3	22.2 ± 8.5
After	24.9 ± 5.7	25.3 ± 6.2	25.8 ± 8.1
P value	0.0009	0.0043	0.0057
Bilirubin (mg/dL)			
Before	0.62 ± 0.09	0.59 ± 0.10	0.61 ± 0.09
After	0.63 ± 0.08 (NS)	0.63 ± 0.08 (NS)	0.64 ± 0.08 (0.028)

Values before and after treatment were analyzed by the two-tailed paired Student's *t* test. Normal values are ALT = 10–44 U/L, AST = 10–34 U/L, and bilirubin = 0.3–1.9 mg/dL. NS = not significant.

Supplemental Table 4

Effects of ARA treatment on plasma urea and creatinine levels of schoolchildren infected with *S. mansoni*

	Mean ± SD before and after treatment with		
	PZQ	ARA	PZQ + ARA
Children	84	87	86
Parameter (mg/dL)			
Urea			
Before	17.5 ± 4.7	16.8 ± 3.7	16.7 ± 4.7
After	19.3 ± 4.6	18.7 ± 4.9	18.1 ± 4.6
P value	0.016	0.002	0.036
Uric acid			
Before	3.3 ± 0.7	3.5 ± 2.6	3.6 ± 2.6
After	3.5 ± 0.6 (NS)	3.5 ± 0.6 (NS)	3.5 ± 0.6 (NS)
Creatinine			
Before	0.57 ± 0.09	0.59 ± 0.09	0.60 ± 0.10
After	0.58 ± 0.10	0.56 ± 0.09	0.58 ± 0.09
P value	NS	0.038	NS

NS = not significant.
